# Predicting overall survival of patients with hepatocellular carcinoma using a three‐category method based on DNA methylation and machine learning

**DOI:** 10.1111/jcmm.14231

**Published:** 2019-02-19

**Authors:** Rui‐Zhao Dong, Xuan Yang, Xin‐Yu Zhang, Ping‐Ting Gao, Ai‐Wu Ke, Hui‐chuan Sun, Jian Zhou, Jia Fan, Jia‐bin Cai, Guo‐Ming Shi

**Affiliations:** ^1^ Department of Liver Surgery and Transplantation Liver Cancer Institute Zhongshan Hospital Fudan University Shanghai China; ^2^ Key Laboratory of Carcinogenesis and Cancer Invasion of Ministry of Education Shanghai China; ^3^ Key Laboratory of Medical Epigenetics and Metabolism Institutes of Biomedical Sciences Fudan University Shanghai China

**Keywords:** DNA methylation, hepatocellular carcinoma, machine learning

## Abstract

Hepatocellular carcinoma (HCC) is closely associated with abnormal DNA methylation. In this study, we analyzed 450K methylation chip data from 377 HCC samples and 50 adjacent normal samples in the TCGA database. We screened 47,099 differentially methylated sites using Cox regression as well as SVM‐RFE and FW‐SVM algorithms, and constructed a model using three risk categories to predict the overall survival based on 134 methylation sites. The model showed a 10‐fold cross‐validation score of 0.95 and satisfactory predictive power, and correctly classified 26 of 33 samples in testing set obtained by stratified sampling from high, intermediate and low risk groups.

## 1 INTRODUCTION

Liver cancer is a life‐threatening malignant disease, and the number of new cases of liver cancer increased by 75% between 1990 and 2015 according to the Global Burden of Disease Study (GBD 2015). In 2015, 854 000 new cases of liver cancer and 810 000 deaths were reported worldwide, making liver cancer the fourth leading cause of cancer‐related death, amounting to a disease burden of 20 578 000 disability‐adjusted life‐years.^1^

Hepatocellular carcinoma (HCC) accounts for 75%‐80% of all cases of liver cancer. The 5‐year overall survival (OS) of HCC patients is 3%‐5% across all countries. Patients with stage A HCC (BCLC) have a 5‐year OS rate of 50%–75%, with different comorbidities.^2^ Increasing evidence suggests that altered or dysregulated DNA methylation may contribute to HCC. DNA methylation plays an important role in regulation of gene expression, development of normal cells and maintenance of tissue stability.^3^ Human DNA methylation occurs only at CpG islands, most of which are located in the promoter and the first exon.^4^ Methylation of the promoter inhibits gene expression, and abnormal methylation is associated with many human diseases, including cancer.^5^ Genomic methylation can be analyzed in a high‐throughput manner, which may facilitate disease diagnosis, prevention and treatment.

In a study of 61 HCC cases, methylation‐specific PCR identified *MLH1, PMS2, MSH2* and *P16* as frequently methylated genes in advanced HCC.^6^ An effective two‐category classification model was generated for predicting early HCC recurrence based on at least three CpG methylation sites; this model was developed through analysis of 450K methylation chip data from 576 publicly available samples.^7^ Analysis of 450K chip data also led to the identification of DNA methylation sites in the genome of peripheral blood mononuclear cells and T cells that were associated with HCC progression.^8^ Bisulfite sequencing analysis in the Huh2 HCC cell line showed an association between abnormal DNA methylation and abnormal *DLL3* expression.^9^ The clinical potential of DNA methylation in HCC was demonstrated when DNA containing methylated *SEPT9* promoter circulating in plasma was found to be a promising biomarker for the disease.^10^

Several studies suggest that DNA methylation may help predict OS of HCC patients. Analysis of 63 HCC samples and 10 normal controls identified methylation sites potentially associated with poor prognosis,^11^ and a study of 27K methylation chip data from 71 HCC patients identified 13 candidate methylation sites. Unfortunately, both studies failed to develop a predictive model because of small sample size.^12^ A larger study of 450K chip data from 304 HCC samples used machine learning to build a model to predict OS based on 36 methylation sites.^13^ Xu et al. analyzed circular tumor DNA methylation sites in 1098 HCC patients and constructed a two‐category classification prognostic model based on 8 DNA methylation sites, which can effectively predict OS of HCC patients.^14^ Yeh et al. analyzed plasma DNA methylation sites in 172 HCC patients and found that LINE‐1 methylation level was significantly correlated with OS, and may be a promising predictor of OS of HCC patients.^15^ The three models established by the above three studies are dichotomy‐based (two categories), making their risk prediction relatively crude. The predicting tool for survival that is based on the molecular information of the patients complements currently existing tumor staging methods that are based on clinicopathologic variables of the patients. Combining these predicting tools and current grading and staging methods will further improve current tumor assessment and guide clinicians to better treatment plan including molecular stratification and risk mitigation, and at the same time offer convenience for data communication among different clinical organizations and further promote research and control on cancer.

Study of the relationship between HCC and DNA methylation is still in its infancy, with relatively few methylation sites associated with HCC prognosis and few predictive models. Here, we used machine learning to analyze DNA methylation data from 450K chips in the TCGA database and to build a model with three risk categories for predicting OS of HCC patients. Our work has implications not only for HCC management but also for other methylation‐associated conditions.

## 2 MATERIALS AND METHODS

### 2.1 Data collection and processing

**Figure 1:**
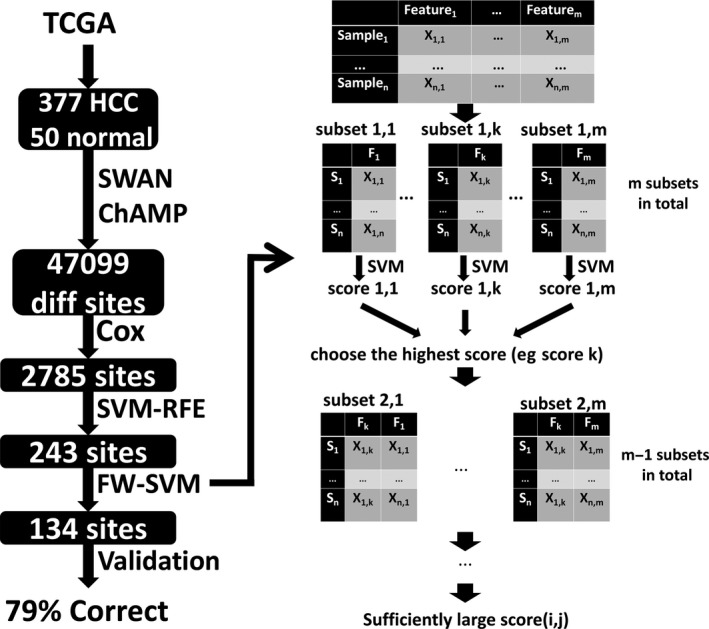
Schematic of the study method. Raw data on DNA methylation of 377 HCC samples and 50 adjacent normal tissue samples based on the Illumina Human Methylation 450 (450K) Bead Chip were downloaded from the TCGA database. By using the ChAMP tool in R software, 40 799 sites methylated differently between HCC tissue and adjacent normal tissue were identified. Then Cox regression was used to assess the potential correlation between OS and each CpG site differentially methylated between HCC and normal tissues. 2785 sites significantly related to OS (*P* < 0.05) were retained. The SVM was then used as a classifier in the SVM‐RFE algorithm to rank features (in our case, methylation sites) from most to least relevant for the training objectives in an iterative process that removes the feature from the background, and the best 243 were selected based on the 10‐fold cross‐validation score for the number of recursive features at each level.The forward‐SVM (FW‐SVM) method was then used to screen feature subsets emerging from the SVM‐RFE analysis. In this process (As shown in the right half of the figure), a model for each feature is constructed, the model with the highest cross‐validation score is selected, and then this feature is combined with each of the others to construct two‐feature models, the best of which is selected based on the cross‐validation score. This process is then iterated to build up multi‐feature models. Finally we built a predictive model containing the best 134 features, and the model was tested using the testing dataset. Of 33 cases, 26 were correctly classified (26/33=79%)

Figure 1 illustrates the study protocol. Raw data on DNA methylation of HCC samples and adjacent normal tissue samples based on the Illumina Human Methylation 450 (450K) Bead Chip were downloaded from the TCGA database. By using the ChAMP tool (version 1.8.2, parameters: differentially methylated sites: *P *< 0.05, |Delta‐Beta| > 0.2) in R software (version 3.2.3), sites methylated differently between HCC tissue and adjacent normal tissue were identified. ChAMP was expressly designed for methylation chips and performs quality control, standardization and calculation of methylation sites and regions.^16^ The beta value was used to estimate methylation levels at CpG loci.

### 2.2 Grouping of patients based on OS

Patients with HCC obtained from the TCGA database were classified as “high‐risk”(58 samples) if they were likely to die within 1 year after surgery; “low‐risk”(41 samples) if they were likely to survive more than 5 years after surgery; and “intermediate‐risk”(64 samples) if they did not fall into either of these two categories. The same three categories were used in the predictive model developed in this study. The median survival of all samples is 416 days (excluding censored patients). In the low‐risk group: the longest follow‐up duration is 3675 days and longest survival is 3258 days. The mean survival is 2525 days for patients who succumbed and 2411 days for those who survived. The high‐risk group and the intermediate‐risk group excluded censored patients, only included non‐surviving patients who fit the definition of “high‐risk” or “intermediate‐risk” in this study. Meanwhile, the low‐risk group included 31 censored patients who were alive at the time of last follow‐up with survival longer than 5 years.

### 2.3 Screening of differentially methylated CpG sites

First, Cox regression was used to assess the potential correlation between OS and each CpG site differentially methylated between HCC and normal tissues. Sites significantly related to OS (*P *< 0.05) were retained. Second, these sites were screened using the Support Vector Machine (SVM)‐Recursive Feature Elimination (RFE) algorithm. The SVM method finds an optimal plane in a multidimensional space that can divide all sample units into two classes, and this plane should maximize the distance between the two nearest points in different classes. The point on the margin between the two nearest points is called the SVM; the split superplane is located in the middle of the space between them. The SVM is then used as a classifier in the SVM‐RFE algorithm to rank features (in our case, methylation sites) from most to least relevant for the training objectives in an iterative process that removes the feature from the background. The SVM‐RFE algorithm may be superior to Linear Discriminant Analysis and Mean Squared Error methods for selecting relevant features and removing redundant features, especially when the number of samples is small.^17^

Third, the forward*‐*SVM (FW‐SVM) method was used to screen feature subsets emerging from the SVM‐RFE analysis. In this process, a model for each feature is constructed, the model with the highest cross‐validation score is selected (see next section), and then this feature is combined with each of the others to construct two‐feature models, the best of which is selected based on the cross‐validation score. This process is then iterated to build up multi‐feature models. The FW‐SVM algorithm is different from SVM‐RFE because it progresses from fewer to more features using a greedy algorithm. The combination of two algorithms can screen features better than either on its own for constructing an SVM model.

Software version and specific implementation: Python 3, *Scikit‐learn (sklearn)* toolkit. *Sklearn* is a Python‐based machine learning module based on the BSD open source license. SVM‐RFE and FW‐SVM mainly utilizes the *svm* module and the *feature_selection* module under the *sklearn* package. The steps of SVM‐RFE are: (a) build the SVM‐RFE model using *RFECV* under the *sklearn.feature_selection* module; (b) use the *fit* function to train the model; (c) obtain the model cross‐validation score by the *cross_val_score* function under the *sklearn.model_selection* module; (d) return the model score for a different number of features, and obtain the final features of SVM‐RFE screening. The FW‐SVM utilizes the same modules as the SVM‐RFE, but finds the best feature set according to the forward recursive process described previously.

### 2.4 Cross‐validation during screening of methylation sites

In each step of the RFE‐SVM and FW‐SVM algorithms, the intermediate and final results were evaluated using the average score obtained from 10‐fold cross‐validation. In cross‐validation, training and testing require multiple iterations of data, and 10‐fold means that the data are randomly divided into 10 batches.^18^ During the next 10 machine learning sessions, each batch was used for validation and the other nine for training. Cross‐validation estimates the error boundary for multiple samples, resulting in a model with lower generalization errors. The mean accuracy of the 10 validation runs was calculated as the 10‐fold cross‐validation score. The closer this score was to 1, the more effective the model was considered.

### 2.5 Model validation and evaluation

The 163 cases of raw data were divided by stratified sampling into a training set (130 cases, 80%) and test set (33 cases, 20%). The SVM model was reconstructed by using the training sample and the final feature combination, and the test samples were used to test the model effectiveness.

## 3 RESULTS

### 3.1 Patient grouping

Raw 450K chip data from 377 HCC samples and 50 adjacent normal tissue samples were downloaded from the TCGA database. Patients who were still alive and for whom fewer than 5 years had passed since surgery were excluded from the analysis. Among the remaining patients, 58 were classified as high‐risk, 64 as intermediate‐risk and 41 as low‐risk.

### 3.2 Identification and screening of differentially methylated sites

**Figure 2:**
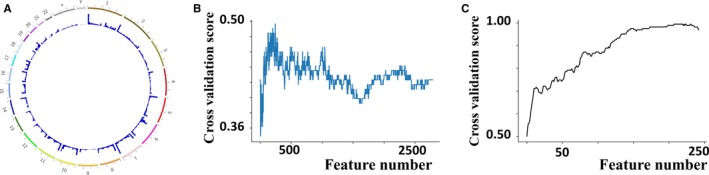
(A) Using ChAMP, we identified 47 099 differentially methylated sites in the sample of 377 HCC samples and 50 adjacent normal tissues. (B) Results of applying the SVM‐RFE algorithm to 2785 methylation sites significantly associated with overall survival based on Cox regression, and the best 243 were selected based on the 10‐fold cross‐validation score for the number of recursive features at each level. The corresponding 10‐fold cross‐validation score was 0.50.C. Results of applying the FW‐SVM algorithm to 243 methylation sites obtained with the SVM‐RFE method, and we finally built a predictive model containing the best 134 features, which gave a mean 10‐fold cross‐validation score of 0.95

Using ChAMP, we identified 47 099 differentially methylated sites in the sample of 377 HCC samples and 50 adjacent normal tissues (Figure 2A). Of these sites, Cox regression identified 2785 differentially methylated sites that correlated significantly with OS (*P *< 0.05). SVM‐RFE was then applied to these 2785 sites, and the best 243 were selected based on the 10‐fold cross‐validation score for the number of recursive features at each level. The corresponding 10‐fold cross‐validation score was 0.50 (Figure 2B).

This score prompted us to perform further screening using the FW‐SVM algorithm, which combined the SVM algorithm with an algorithm that progressively filters feature subsets forward. In order to obtain the “best model with the fewest features”, we built a predictive model containing the best 134 features, which gave a mean 10‐fold cross‐validation score of 0.95 (Figure 2C).

### 3.3 Model validation

The SVM model was reconstructed using the training dataset and 134 feature combinations, and the resulting model was tested using the testing dataset (Table 1). Of 33 cases, 26 were correctly classified. These results suggest that the model can effectively predict OS of HCC patients on the basis of methylation status without over‐fitting (Table 2). To further validate the predictive power of the model, we tested it on 19 paired HCC and normal tissue samples from the GSE77269 dataset in the GEO database. The model did not classify any samples as “high‐risk” in normal adjacent liver tissues, but classified 7 samples as “high‐risk” in 19 HCC tissues, and this ratio (7/19 = 36.8%) is very consistent with that in the TCGA database (58/163 = 35.8%).

**Table 1:** Stratified sampling of patients based on overall survival after surgery

Risk group	Patients in dataset (n)	
	Training	Testing
High	46	12
Intermediate	51	13
Low	33	8

**Table 2:** Model validation

Predicted/Actual	High risk	Intermediate risk	Low risk
High risk	12	0	0
Intermediate risk	2	9	2
Low risk	1	2	5

## 4 DISCUSSION

Here we achieved a reasonable predictive model of OS in HCC patients based on 134 methylation sites. This number of features is not particularly large for general machine learning, but it is still too many for optimal performance in molecular biology tasks. The relatively large number of features reflects the relatively small sample of 163 cases, which in turn reflects the fact that much of the TCGA data did not satisfy classification requirements. This makes feature selection difficult and increases the risk of model over‐fitting. Fortunately, our model was able to predict reasonably well without over‐fitting. This illustrates how the combination of the SVM‐RFE and FW‐SVM algorithms can be effective when the number of samples is small. We speculate that this is because compared to SVM‐RFE, the FW‐SVM is closer to the exhaustive algorithm (traversing all feature combinations to identify the features that perform best on the test set), which can finely screen the features, so there is generally better performance in the final stage of model training. In this study, the number of initial features was large and the number of samples was small. As a result, the SVM‐RFE algorithm score is not high, and the FW‐SVM algorithm has a better improvement on the results of the previous step, which is within our expectation. Integrating more sample data would doubtlessly allow us to generate a model with fewer features. Data mining of the growing methylation database will continue to shed insights on the association between DNA methylation at specific sites and HCC phenotypes.

Here, we compare the model obtained in this study with other DNA methylation‐based survival prediction models for HCC. Many researchers have investigated prognostic predictors of HCC based on methylation sites. Compared with these models or molecular biomarkers, the advantage of our model is that it is a three‐category model with a satisfactory accuracy of prediction. The former two‐category classification models usually did survival analysis of high‐risk and low risk patients in the validation set to judge the effectiveness of the model. Three‐category classification model is more detailed than two‐category classification models if its accuracy is acceptable. Here, we take a recent representative study by Xu et al. mentioned previously as an example for comparison with our study: Xu, et al. analyzed circular tumor DNA methylation sites in 1098 patients with HCC and constructed the two‐category classification prognostic model based on 8 DNA methylation sites, combined prognosis score (cp‐score), classified samples with survival data into the high‐risk group and the low‐risk group. Kaplan‐Meier curves showed significant difference of prognosis between the two groups in the validation set, log‐rank test *P* = 0.0014, hazard ratios [HR] (high‐risk vs. low‐risk) = 3.13, CI:1.64‐6.25, *P *< 0.0001. If our model is analyzed by the same method, our model classified the test set into three groups: the high‐risk group, the intermediate‐risk group and the low‐risk group. Here are five comparisons: (a) high‐risk vs. low‐risk: log‐rank test *P *< 0.001, HR = 8.95 (1.96‐40.92), *P *= 0.005; (b) High‐risk vs. medium‐risk: log‐rank test *P* = 0.007, HR = 3.12 (1.31‐7.46), *P *= 0.01; (c) Medium vs. low‐risk: log‐rank test *P *= 0.04, HR = 4.58 (0.97‐21.64), *P *= 0.055; (d) High‐risk vs. (medium‐risk + low‐risk): log‐rank test *P *< 0.0001, HR = 4.77 (2.09‐10.90), *P *= 0.0002; (e) (high‐risk + medium‐risk) vs. low‐risk: log‐rank test *P* = 0.004, HR = 6.41 (1.49‐27.54), *P* = 0.01. In conclusion, our model is able to classify test sets effectively and yet in greater details compared with the two‐category classification models.

It should be noted that in addition to the small number of samples, we still face some problems when using TCGA data. The samples of TCGA were collected mainly from the US, which brings about the following problems: in terms of sample ethnicity, the samples of TCGA are mainly from Caucasians; in terms of the underlying diseases of HCC, the cause of HCC varies significantly among countries. Furthermore, in terms of the overall quality of medical care: the distribution of OS in our study is different from other countries, possibly due to better health care condition in the US.

After the final model was established, we carried out gene annotation enrichment analysis of the detected marker genes and found that these genes were highly enriched in such biological processes as regulation of transcription from RNA polymerase II promoter, apoptosis, and angiogenesis, and cellular components including lysosomal membrane. These biological processes and components play an important role in oncogenesis and cancer progression.

Emerging new technologies such as in genomics and proteomics provide new approaches for exploration of novel diagnostic and prognostic biomarkers of HCC, including DNA, mRNA, microRNA, proteins, metabolites, and abnormally methylated DNA. The developing algorithmic technologies also offer tremendous help in the birth of new prediction models, especially machine learning, including Deep Learning, Decision Tree and SVM. In the machine learning model of HCC diagnosis and prognosis, many representative studies have appeared in recent years. Omran et al. (2015) constructed the decision tree model to predict prognosis of HCC patients based on the clinical data of 315 HCV patients, 116 liver cirrhosis patients, and 135 HCC patients, yielding a sensitivity of 83.5% and an accuracy of 83.3%.^19^ Wang et al. (2015) constructed the decision tree model to predict post‐hepatectomy liver failure of HCC patients based on the surgical data of 634 HCC patients.^20^ Cao et al. (2013) trained the decision tree by serum protein spectrum of 50 post‐hepatectomy patients with HCC, and then used 36 homogeneous patients to validate the accuracy of the decision tree. They found that the serum biomarkers could predict post‐hepatectomy intrahepatic recurrence of HCC patients.^21^ Ho et al. (2012) used SVM algorithm and neural network to train the machine learning model based on the clinical data of 482 cases that received HCC resection, in order to predict recurrence and survival. Moreover, Ho et al. judged the merits and drawbacks of their models by comparing the area under the ROC curve in different models.^22^ Augello et al. (2018) found that two SNPs rs2596542 and rs2596538 of the MICA gene and “age” could be used for identification and classification of liver cirrhosis and HCC by using sorting algorithm in machine learning.^23^ Chandhary et al. (2018) constructed a multi‐layer artificial neural network model containing three hidden layers based on RNA sequencing, miRNA sequencing and methylation data of 360 samples in TCGA, and further determined subgroup classification of HCC patients by survival.^24^ Liang et al. (2016) combined machine learning and metabonomics and identified 15 metabolites in urine; these metabolites are involved in several critical metabolic pathways and could differentiate HCC patients from normal subjects. Five of the metabolites are of diagnostic value for HCC with a sensitivity of 96.5% and an accuracy of 83%.^25^ As shown previously, new algorithms and biomolecular techniques have been applied for constructing HCC diagnostic and prognostic models. The continuously developing technologies have brought about massive data, yet we are still not able to understand and analyze these data profoundly. Since the current models only contain limited variations, it will be an exciting research area to construct a predicting model that not only takes full advantage of patients’ clinicopathologic data but also contains multi‐level molecular data.

## CONFLICT OF INTEREST

The authors declare that they have no conflict of interest.
